# Muscle Coactivation Index during Walking in People with Multiple Sclerosis with Mild Disability, a Cross-Sectional Study

**DOI:** 10.3390/diagnostics13132169

**Published:** 2023-06-26

**Authors:** Francisco Molina-Rueda, Diego Fernández-Vázquez, Víctor Navarro-López, Raúl López-González, María Carratalá-Tejada

**Affiliations:** 1 Physical Therapy, Occupational Therapy, Rehabilitation and Physical Medicine Department, Faculty of Health Sciences, Rey Juan Carlos University, 28922 Madrid, Spain; francisco.molina@urjc.es (F.M.-R.); diego.fernandez@urjc.es (D.F.-V.); maria.carratala@urjc.es (M.C.-T.); 2 Movement Analysis, Biomechanics, Ergonomics, and Motor Control Laboratory, Faculty of Health Sciences, Rey Juan Carlos University, 28922 Madrid, Spain; gonzalez.raul@urjc.es

**Keywords:** gait, multiple sclerosis, muscle coactivation

## Abstract

Background: Multiple sclerosis (MS) is a progressive neurodegenerative disease characterized by axonal degeneration and demyelination. Changes in gait, related to joint kinematics and kinetics, especially at the ankle and knee, have been observed in people with MS (pwMS). Muscle coactivation plays an important role in joint stabilization; however, excessive coactivation may interfere with gait. The aim of this study was to analyze the differences in muscle activation during gait in pwMS compared to healthy individuals. Methods: A cross-sectional study was conducted involving pwMS and healthy controls. Surface electromyography was used to record muscle activity during gait. The main outcome measures were the coactivation index (CI) and the area under the curve (AUC), which were calculated for several pairs of lower extremity muscles. Results: Nine pwMS and nine healthy controls were included. When comparing the MS group to the control group, the AUC was significantly higher in the lateral gastrocnemius (*p* = 0.023) and the CI for the lateral gastrocnemius-anterior tibialis (*p* = 0.022) and gluteus maximus-lateral gastrocnemius (*p* = 0.047). Conclusion: Mildly affected pwMS have altered muscle coactivation patterns during gait, especially in the most affected limb. The results highlight the importance of muscle coactivation in pwMS and its possible role in the early detection of gait abnormalities.

## 1. Introduction

Multiple sclerosis (MS) is a progressive neurodegenerative disease of the central nervous system caused by axonal degeneration and demyelination [1]. The immune system mistakenly attacks and damages tissue in the brain, spinal cord and optic nerve and people with early MS already experience brain atrophy more rapidly than the general population [2]. People with MS (pwMS) usually have gait abnormalities that increase their disability. Approximately 75% of pwMS have been reported to have clinically significant impairments, even those with mild disability in the early stages of the disease [1]. Their gait deficit is a major contributor to a reduced quality of life, reduced activities of daily living and the loss of employment [3]. PwMS walk with reduced hip extension and hip extension moment in the stance phase. At the knee, there is some consensus that pwMS have reduced flexion during swing. At the ankle, the main changes described are reduced dorsiflexion during stance and limited plantar flexion during toe off [4]. Molina-Rueda et al. (2022) showed a symmetrical gait pattern in pwMS with mild disability [5]. However, the authors reported that, in these patients, the temporal occurrence of kinematic and kinetic parameters, expressed as a percentage of the gait cycle, was delayed. On the other hand, other studies have observed changes in joint kinematics and kinetics in pwMS with mild disability [6,7,8,9,10]. Kempen et al. reported that pwMS have reduced ankle push off as an important contributor to impaired gait performance [11]. Eken et al. showed evidence of soleus muscle fatigue in pwMS during prolonged walking [1].

Surface electromyography (sEMG) of gait has applications in the diagnosis of conditions such as abnormal load response [12]. Muscle coactivation is the simultaneous contraction of agonist and antagonist muscles across a joint [13]. During a single joint movement, an antagonist muscle is inhibited to allow an agonist muscle to move smoothly; this is known as reciprocal inhibition. During skilled movements, young people produce a net torque at the joint by activating agonist and antagonist muscles through optimal scaling [14]. Muscle coactivation stabilizes the joint during skilled motor performance.

Several authors [15,16] have reported that people with neurological conditions have increased coactivation of agonist–antagonist muscles around the ankle joint. Coactivation stabilizes joints [17], but excessive coactivation can reduce net torques and increase stiffness [18]. In this sense, Ervilha et al. used the coactivation index (CI) to analyze the sEMG of the contralateral muscles [19]. Iwamoto et al. showed a greater muscle co-contraction of the ankle joint during dynamic postural control in elderly subjects than in young subjects, not only in the preferred speed condition but also in the fast speed condition [20].

Even though adaptive brain plasticity can occur in pwMS, the accumulation of neurological damage may lead in muscle impairments as an increased muscle coactivation [21]. Few studies have investigated the neuromuscular strategies that pwMS with mild disability use to cope with their impairments. Boudarham et al. [22] showed increased agonist–antagonist coactivation in the knee muscles during single support and in the ankle muscles during double support in pwMS with an EDSS score of 5 or less compared with healthy subjects. The authors hypothesized that this increase in coactivation could be a compensatory mechanism to limit the risk of falling due to altered motor control, or it could be because pwMS walk at a significantly lower speed than healthy subjects [22]. There is a need for more articles investigating muscle coactivation in pwMS who have mild involvement. In addition, it is necessary to analyze the coactivations between muscles of different joints, as these are activated by creating synergies during walking. We hypothesized that pwMS would have reduced muscle activity and increased coactivation of muscles during regular walking. This study has the potential to monitor pwMS for the early detection of gait impairment before kinematic and clinical changes in gait are detectable.

## 2. Materials and Methods

### 2.1. Design

A cross-sectional study was conducted in pwMS to analyze the differences in muscle activation during gait. The local university research ethics committee approved the study and informed consent was obtained from all participants.

### 2.2. Participants

Written informed consent was obtained prior to all procedures, which were conducted in accordance with the tenets of the Declaration of Helsinki and the Biomedical Research Act 14/2007.

Both pwMS and control subjects were asked to participate on a voluntary basis, with recruitment starting in September 2022 and ending in January 2023. Patients had to fulfil the following inclusion criteria: (1) aged over 18 years, (2) diagnosed with MS based on the 2017 revised McDonald criteria [23], (3) EDSS score less than or equal to 3 [24], (4) presence of other comorbidities (e.g., musculoskeletal, cardiorespiratory, rheumatic, or other neurological diseases than MS). Participants were excluded if they had experienced a worsening of symptoms, required hospitalization, intravenous or oral corticoid therapy, botulinum toxin or any other situation that could potentially affect their participation in the study in the previous six months. 

Healthy controls were recruited from family contacts surrounding the pwMS. Inclusion criteria for the matched control subjects were: (1) aged over 18 years, (2) the ability to walk independently without assistance, (3) and the absence of musculoskeletal and/or neurological disorders.

### 2.3. Procedure

The experimental protocol was carried out according to the STROBE checklist [25]. The participants were evaluated at the Laboratory of Movement Analysis, Biomechanics, Ergonomics and Motor Control, located at the Faculty of Health Sciences, Rey Juan Carlos University. Surface electromyographic (sEMG) activity during gait was recorded in the pwMS and in the control group. The muscles recorded by sEMG from both legs (bilaterally) during walking were the gluteus maximus (GM), gluteus medius (GMED), rectus femoris (RF), biceps femoris (BF), tibialis anterior (TA) and the lateral head of the gastrocnemius (LG). The sEMG was assessed using the Cometta Zero^®^ wireless EMG system (Bareggio, Italy). The skin was shaved and cleaned with alcohol where the electrodes were to be placed. For each muscle, two adhesive Ag/AgCl electrodes were placed according to the SENIAM protocol [26]. A manual muscle test was performed to verify the correct positioning of the electrodes. sEMG signals were recorded at a sampling frequency of 1000 Hz. Both lower limbs were recorded; the more affected limb was defined according to the medical evaluation. After instrumentation, subjects were asked to walk through the 10-m corridor laboratory at a self-selected comfortable walking speed, with a minimum of five repetitions recorded per subject. Only the central 5 m were recorded to avoid the effect of gait acceleration and deceleration. To avoid potential bias, the evaluator who processed the gait tests was independent of the researcher responsible for analyzing the results. The same protocol was used for both study groups.

### 2.4. Outcome Measures

The main assessments used to compare the two groups, pwMS and controls, were the CI and the area under the curve (AUC). CI is defined as the mathematical formula calculated when applying coactivation analysis. The equivalent formula is given in this expression [16,20,27].

(1) $$\mathrm{CI}=\frac{\mathrm{Overlapping}\;\mathrm{area}\;\mathrm{of}\;\mathrm{agonist}\;\mathrm{and}\;\mathrm{antagonist}\;\mathrm{muscles}\;}{\mathrm{Area}\;\mathrm{of}\;\mathrm{agonist}\;\mathrm{muscle}+\mathrm{Area}\;\mathrm{of}\;\mathrm{antagonist}\;\mathrm{muscle}}\left(X\right)$$

The corresponding values for the areas mentioned above were assessed by calculating the AUC of the following pairs of muscles: biceps femoris and rectus femoris (BF–RF); lateral gastrocnemius and tibialis anterior (LG–TA); gluteus maximus and rectus femoris (GM–RF); gluteus medius and gluteus maximus (GMED–GMAX); gluteus maximus and lateral gastrocnemius (GMAX and LG). These muscle pairs have been selected according to the sequence of muscle activation during the gait cycle, during which they either co-activate by stabilizing a joint or by creating a synergy to ensure gait functions (heel strike absorption and progression) [28].

**Figure 1:**
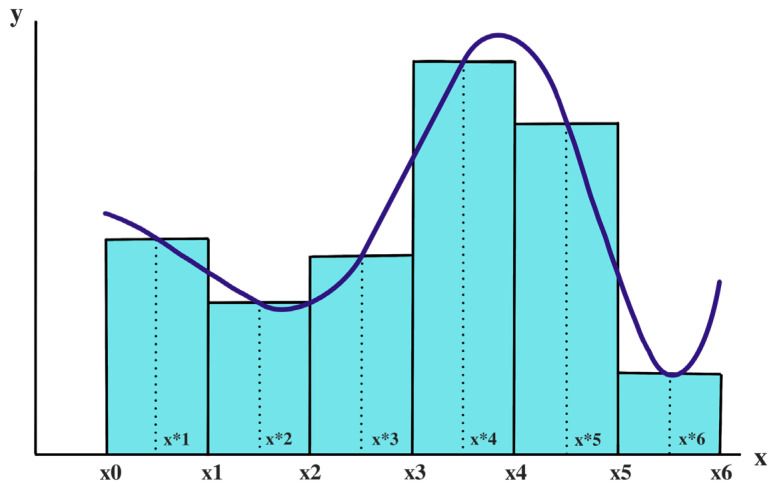
Visual example that represents the functionality of the ‘trapz’ function extracted from Python. [x0, …, x6] are the values along the *x*-axis. *Y*-axis implies the amplitude value for each of the points mentioned before. The corresponding curve will be integrated by approximating them into trapezoid shapes as the blue rectangles of the figure.

The tool used to obtain the AUC of each subject was “trapz”, a function extracted from the Python library called NumPy, which is a high-level programming language. This application has the function of integrating any input along the *x*-axis if it is provided [29]. If this is not the case, the sample reference points are assumed to be equally spaced dx apart. The method used by this tool to obtain the result is the trapezoidal rule. This method implies that the solution of the operation is obtained by approximating the definite integral to a region such as a trapezoid, so that its value can be calculated as an area. A more visual way of understanding this concept is to compare it to the estimation of the area of a given geometric figure, in this case a trapezium, with a specific base that goes from 0 to 100 (equivalent to the gait cycle). The fact that this module gives different results depending on the muscle and/or subject simply means that the shape of the approximated trapezoid has greater or lesser dimensions (Figure 1).

### 2.5. Data Analysis

The recordings obtained from the VICON system were directly processed using libraries from a high programming language, Python, to complete the pre-processing and finally the metrics. As a first step, the sEMG was filtered using two types of filters. In one, a 20 Hz second order high-pass filter. The other was a 250 Hz fourth order low-pass filter. Both cut-off frequencies were discussed by the team after research and looking at different results, so that standard values could be set. However, an extra option was added to allow for manual filtering by selecting different values from those mentioned above in case a subject required individual filtering rather than automatic filtering [16,20,30,31]. To complete the pre-processing part, the signals obtained after filtering were rectified to discriminate negative values. As a final step, we tried to obtain the linear envelope so that the output signal would be as smooth as possible. The method used was based on the convolution of a scaled window with the signal.

The signal was prepared by introducing reflected copies of the signal (with a window size of 100 ms) at both ends, so that the transient parts at the beginning and end of the output signal were minimized. The mean amplitude of the RMS sEMG signals was calculated for the gait cycle. sEMG amplitude, frequency and duration are influenced by many factors such as muscle fiber type, speed of action, subcutaneous fat thickness and electrode placement. To reduce between-subject and between-experiment differences, amplitude normalization was performed based on the maximum peak of the RMS during gait for each individual and each muscle separately. Normalization to the peak or mean amplitude during the activity of interest has been shown to reduce variability between individuals compared to using raw EMG data or normalizing to maximum voluntary isometric contractions [32].

### 2.6. Sample Size Determination

We calculated the sample size to detect a minimum difference of 20 between two groups, assuming 3 groups and a standard deviation of 10, based on the results of Boudarham al. 2016 [22]. We accepted an alpha risk of 0.05 and a beta risk of 0.1, in a bilateral contrast, and a loss-to-follow-up rate of 20%. Based on this calculation, 9 subjects were required in each group. The GRANMO sample size calculator was used to calculate the sample size [33].

### 2.7. Statistical Analysis

Statistical analyses were performed with the SPSS program (version 27.0; IBM Corp., Armonk, NY, USA). The Shapiro–Wilk test was performed, which showed that the distribution of all variables included followed a normal distribution.

One-way analysis of variance (ANOVA) with Bonferroni post hoc adjustment was used to compare the more affected lower limb of the MS subjects, the less affected lower limb of the MS subjects and the control limb (dominant lower limb of the healthy subjects). Student’s *t*-test for independent samples was used to compare participant characteristics and walking speed. Effect sizes were obtained for all comparisons (η²). A significance level of 0.05 was used for all statistical comparisons.

## 3. Results

A total of nine pwMS and nine age- and sex-matched healthy controls were included in this study. The demographic characteristics of the subjects included in the study are shown in Table 1. Table 2 shows the CI and AUC values for the different muscles. No significant differences were observed in the main demographic data studied, and the participants presented similar anthropometric characteristics and spatiotemporal gait parameters. The mean (SD) EDSS score of the subjects with MS was 1.83 (1.2), ranging from 0 to 3. All patients included had relapsing-remitting MS. The left hemisphere was the most affected hemisphere in the MS subjects (77%), whereas the right hemisphere was dominant in 100% of the healthy controls.

The results show a greater AUC in the activations of the GM, GMED, BF, RF, LG, and TA muscles in the most affected lower limb of pwMS compared to controls. However, one-way ANOVA only showed a significant effect of group on the AUC LG (F = 4.52; *p* = 0.023). Specifically, post hoc analysis showed that AUC LG was higher in the more affected lower limb of pwMS compared to the less affected lower limb and controls (Table 3).

For the CI, the results showed higher values in the more affected lower limb of pwMS compared to controls in the following muscle groups: GMED and GM, BF and RF, LG and TA, and GM and LG. These results are similar in the less affected lower limb compared to controls. However, statistical analysis showed significant results only in the LG and TA pairs (F = 4.460; *p* = 0.024) and the GM and LG pairs (F = 4.165; *p* = 0.031). Post hoc analysis showed significant differences between the more affected lower limbs of pwMS and controls in the muscles described (Table 4).

**Figure 2:**
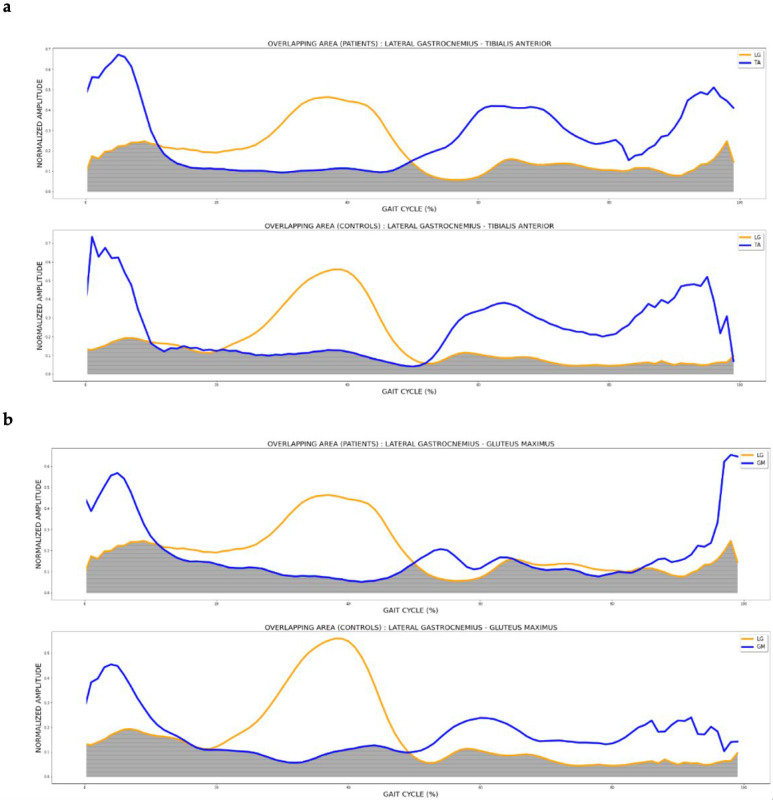
Overlapping areas between (**a**) LG and TA; and (**b**) GM and LG in patients (more affected lower limb) and controls (dominant lower limb). The grey shading shows the overlapping between muscle activations.

Figure 2 shows the overlapping area (grey area) of the lateral gastrocnemius and tibialis anterior muscles during the gait cycle in pwMS (most affected side) and controls. A larger area was observed in patients.

## 4. Discussion

In this cross-sectional study, we identified changes in the coactivation of the main muscles involved in gait in mildly disabled pwMS compared with age-matched healthy controls. The patients in our study had relatively short disease duration and low EDSS scores, and all walked independently without the use of assistive devices, showing a self-selected walking speed similar to that observed in other studies [33,34]. The EDSS scores shown by these patients were <3. Scores between 1.0 and 4.5 refer to individuals with independent walking ability, but who present objective alterations in imaging tests in certain functional systems of the central nervous system, such as: pyramidal, cerebellar, brainstem, sensory, bowel and bladder, or visual [35]. PwMS with mild disability showed changes in the coactivation rates of the lateral gastrocnemius with the tibialis anterior and the gluteus maximus with the lateral gastrocnemius, with greater coactivation in the most affected leg during the gait cycle compared to age- and sex-matched healthy controls.

Muscle coactivation occurs during the gait cycle to achieve joint stability; for example, during the loading response phase, biceps femoris and rectus femoris coactivation occurs. However, an increase in coactivation during gait may be the response to a lack of joint control, so that coactivation compensates for poor control [18,36]. This adaptation may occur to help maintain postural stability, thus preserving gait. Our findings have been similar to those found by Boudarham et al. [22] who observed increased coactivation in the knee muscles during single support, and in the ankle muscles during double support, in pwMS with a mean EDSS score of 3.8. These authors observed that there is a higher CI in the patients with the slowest gait, the greatest motor impairment and the most instability; this is related to the fact that in our study we only observed differences in the CI of the most affected leg. Based on these findings, muscle coactivation indices during gait may be useful in the early diagnosis of dynamic postural alterations, with a progression in these being observed as the disease progresses [37].

The increased coactivation of the tibialis anterior and lateral gastrocnemius, muscles that, in the absence of pathology, perform an agonist–antagonist activation during the gait cycle, seems particularly relevant [38]. The increased coactivation in these muscles, as presented by the patients evaluated in this study, compared to healthy controls, may be the response to a lack of stability in the ankle and knee joints. These findings may be related to those found in pwMS with mild disability who did not show gait disturbances on observational analysis, who showed delayed take-off, and delayed kinetic and kinematic events at the ankle and knee joint, but not at the hip [5].

Furthermore, the results of our work showed a coactivation between the lateral gastrocnemius and the gluteus maximus during the gait cycle. These muscles are normally activated during the stance phase, contributing to propulsion and progression of the lower limb [28]. Under certain circumstances, such as inclined gait, there is increased muscle work of the hip extensors and plantar flexors in the stance phase, mainly to generate sufficient propulsion to accelerate the center of mass forward and upward [39]. The presence of increased coactivation between the gluteus maximus and lateral gastrocnemius muscle highlights the need for increased stabilization during gait in pwMS who are mildly disabled.

The clinical implications that increase coactivation between muscles in MS will depend on when in the gait cycle they occur. According to the description followed by Boudarham et al. [22]., if increased coactivation occurs at the time of double stance, an increased stance phase may occur, which is related to impaired dynamic balance [40,41]. Excessive coactivation in the ankle, as observed in this study, could be related to a neuromuscular system to improve mechanical stability. Increased coactivation is intended to stabilize the joint by reducing the degree of movement [42], and may improve the load-receiving phase, and weight transfer to the contralateral limb. Regarding muscle coactivation in the monopodal stance phase, Boudarham et al. [22] showed increased coactivation of the knee muscles, but decreased coactivation of the muscles controlling the ankle joint in pwMS and low disability. This group of authors hypothesized that increased coactivation of the hip muscles could exert the function of replacing the lost stability in the ankle with the decreased coactivation presented by the tibialis anterior and lateral gastrocnemius. In our study, we did not perform a phase separation of double stance or monopodal stance, so the patients included in our study may show a similar behavior. It is possible that, regardless of when muscle coactivation occurs, it may lead to an increase in energy expenditure during walking [43]. Furthermore, if the degree of coactivation is greater in the more advanced stages of the disease, this could have a major impact on fatigue, which is relevant and prevalent in MS.

In other neurological pathologies with motor disturbances affecting gait and balance, the findings are similar. Lamontagne et al. [44] observed similar increases in coactivation indices in people after stroke, with greater coactivation in patients with greater disability. Rinaldi et al. [43] observed that higher rates of coactivation correlate with higher scores on the Ashworth scale in people with hereditary spastic paraparesis. In the early stages of Parkinson’s disease, increased coactivation rates of the tibialis anterior and lateral gastrocnemius were similarly observed. The increased muscle coactivation may be related to a poor ability to inhibit the antagonist and modulate its muscle activities [16]. 

Excessive coactivation, as well as inadequate muscle activity during gait, could be relevant to the onset of fatigue, in relation to the increased energy cost during gait, being of special interest in pwMS. Different groups of authors suggest this is an association between the energetic cost of locomotion and muscle activity during gait in pwMS [45,46,47] but there is a lack of studies to evaluate this association objectively. In addition, it has been observed that in pwMS, with mild or no disability, lower stability during gait is associated with an axonal loss of the corticospinal tract, being a possible sensitive indicator of neurodegeneration [48]. Studies should be developed to assess how coactivation rates during gait may influence the increase in energy cost and the onset of fatigue during gait, and whether increased coactivation rates are related to the progression of MS.

This study has several limitations. Because our sample of pwMS was rather small, caution should be exercised in generalizing the results. Therefore, future studies with a larger sample and with comparable groups in terms of gender and age are needed. Another limitation was the variability of the data. In our study, the standard deviation for some parameters was very high. Finally, it would have been more appropriate to analyze the coactivations according to the phases of the gait. However, the entire gait cycle was used to determine whether coactivation between muscles could be detected in a general way, without specifying the phases where the results may be more obvious. This finding suggests that muscle coactivation during the gait cycle may be a sensitive parameter for detecting early gait impairment in pwMS.

## 5. Conclusions

PwMS with mild disability (EDSS ≤ 3) have greater muscle activation during gait in the most affected lower limb compared with subjects without pathology. In addition, pwMS have a higher percentage of muscle coactivation, mainly between the TA–LG and GMAX–LG muscles during the gait cycle.

## Figures and Tables

**Table 1:** Characteristics of participants.

Parameters	Group	Mean (SD)	*p* Values *
Age (years)	MS	34.44 (8.90)	0.958
	Control	34.67 (8.80)	
Sex (female %)	MS	77.78	1.000
	Control	77.78	
Weight (Kg)	MS	68.63 (9.15)	0.278
	Control	63.21 (11.21)	
Height (cm)	MS	169.60 (0.09)	0.448
	Control	172.50 (0.81)	
Cadence (steps/min)	MS	113.85 (8.33)	0.621
	Control	112.12 (6.07)	
Walking speed (m/s)	MS	1.19 (0.15)	0.417
	Control	1.25 (0.12)	
Foot off (%)	MS	61.43 (2.12)	0.275
	Control	60.38 (1.81)	
Stride length (m)	MS	1.26 (0.10)	0.108
	Control	1.35 (0.10)	
ME type (RR)	MS	100%	
EDSS	MS	1.83 (1.20)	
Years since diagnosis	MS	6.9 (5.7)	

The mean (SD) of the main demographic and clinical characteristics is presented. Differences were evaluated using Student’s *t* test for independent samples. M: meters; MS: multiple sclerosis. RR: relapse remitting; S: seconds. * Significant at *p* < 0.05.

**Table 2:** CI and AUC values.

Lower Limb	AUC BF	AUC RF	AUC GastrocL	AUC TA	AUC Gmed	AUC Gmax
Control	17.08 (4.67)	24.83 (7.13)	18.22 (1.24)	21.64 (1.48)	14.14 (0.38)	15.65 (3.06)
MS MALL	18.37 (5.93)	23 (4.18)	22.42 (1.43)	28.12 (1.25)	17.84 (4.26)	15.71 (2.17)
MS LALL	19.83 (3.57)	15.06 (6.26)	18.6 (3.08)	24.13 (4.41)	15.99 (3.64)	14.76 (4.43)
Lower Limb	CI (%) BF-RF	CI (%) GastrocL–TA	CI (%) Gmax–RF	CI (%) Gmed–Gmax	CI (%) Gmax–GastrocL	
Control	53.55 (8.80)	36.44 (7.46)	54.59 (9.34)	55.8 (12.2)	40.19 (6.64)	
MS MALL	51.63 (5.64)	54.05 (13.73)	58.04 (6.38)	68.29 (5.94)	51.53 (8.39)	
MS LALL	57.54 (12.16)	41.57 (10.10)	66.44 (12.6)	69.97 (9.69)	40.81 (12.27)	

The means (SD) of the CI and AUC values of the dominant lower limb of healthy controls and of the most affected lower limb (MALL) and less affected lower limb (LALL) legs of pwMS are shown.

**Table 3:** Results for AUC muscle activations.

Dependent Variable	ANOVA			Post Hoc Analysis (Bonferroni)			
	F	*p* Value	η²	Group	DM	*p* Value	95%CI
GM	0.875	0.430		Control vs. MS MALL	−0.21	1.000	−5.50 to 5.08
			0.071	Control vs. MS LALL	2.19	0.844	−2.94 to 7.33
				MS LALL vs. MS MALL	2.40	0.758	−2.88 to 7.70
GMED	0.761	0.478		Control vs. MS MALL	−0.75	1.000	−4.94 to 3.43
			0.062	Control vs. MS LALL	1.22	1.000	−2.84 to 5.28
				MS LALL vs. MS MALL	1.97	0.709	−2.21 to 6.16
BF	0.514	0.605		Control vs. MS MALL	−1.10	1.000	−6.33 to 4.12
			0.045	Control vs. MS LALL	−2.04	0.966	−7.27 to 3.18
				MS LALL vs. MS MALL	−0.93	1.000	−5.82 to 3.95
RF	1.708	0.203		Control vs. MS MALL	−0.71	1.000	−8.70 to 7.27
			0.129	Control vs. MS LALL	4.47	0.449	−3.27 to 12.23
				MS LALL vs. MS MALL	5.19	0.321	−2.79 to 13.18
LG	4.520	0.023 *		Control vs. MS MALL	−4.73	0.020 *	−8.83 to −0.62
			0.191	Control vs. MS LALL	−1.91	0.677	−5.90 to 2.06
				MS LALL vs. MS MALL	2.81	0.243	−1.17 to 6.79
TA	1.516	0.241		Control vs. MS MALL	−3.19	0.291	−7.95 to 1.57
			0.117	Control vs. MS LALL	−1.99	0.869	−6.76 to 2.76
				MS LALL vs. MS MALL	1.19	1.000	−3.42 to 5.81

DM is the difference of means between groups. CI is the 95% confidence of interval. Differences were evaluated using one-way ANOVA with Bonferroni post hoc analysis. BF: biceps femoris, GM: gluteus maximus, GMED: gluteus medius, LALL: less affected lower limb, LG: lateral head of the gastrocnemius, MALL: more affected lower limb, MS: multiple sclerosis, η²: eta square, RF: rectus femoris, TA: tibialis anterior. * Significant at *p* < 0.05.

**Table 4:** Results for CI between muscle pairs.

Dependent Variable	ANOVA			Post Hoc Analysis (Bonferroni)			
	F	*p* Value	η²	Group	DM	*p* Value	95%CI
GM–GMED	1.640	0.218		Control vs. MS MALL	−6.60	0.625	−19.85 to 6.63
			0.135	Control vs. MS LALL	−8.76	0.275	−21.66 to 4.13
				MS LALL vs. MS MALL	−2.15	1.000	−14.59 to 10.27
BF–RF	0.707	0.503		Control vs. MS MALL	−2.28	1.000	−14.52 to 9.94
			0.058	Control vs. MS LALL	−5.44	0.745	−17.31 to 6.42
				MS LALL vs. MS MALL	−3.15	1.000	−15.39 to 9.07
LG–TA	4.460	0.024 *		Control vs. MS MALL	−16.82	0.022 *	−31.56 to −2.09
			0.298	Control vs. MS LALL	−7.57	0.552	−21.92 to 6.76
				MS LALL vs. MS MALL	9.25	0.290	−4.58 to 23.08
GM–RF	1.228	0.313		Control vs. MS MALL	2.34	1.000	−12.03 to 16.71
			0.105	Control vs. MS LALL	−5.54	0.944	−19.53 to 8.45
				MS LALL vs. MS MALL	−7.88	0.431	−21.37 to 5.61
GM–LG	4.165	0.031 *		Control vs. MS MALL	−15.70	0.047 *	−31.78 to −0.38
			0.294	Control vs. MS LALL	−1.20	1.000	−16.36 to 13.95
				MS LALL vs. MS MALL	14.49	0.064	−0.66 to 29.65

DM is the difference of means between groups. CI is the 95% confidence of interval. Differences were evaluated using one-way ANOVA with Bonferroni post hoc analysis. BF: biceps femoris, GM: gluteus maximus, GMED: gluteus medius, LALL: less affected lower limb, LG: lateral head of the gastrocnemius, MALL: more affected lower limb MS: multiple sclerosis, η²: eta square, RF: rectus femoris, TA: tibialis anterior. * Significant at *p* < 0.05.

## References

[1] Eken M.M., Richards R., Beckerman H., van der Krogt M., Gerrits K., Rietberg M., de Groot V., Heine M. (2020). Quantifying muscle fatigue during walking in people with multiple sclerosis. Clin. Biomech..

[2] Giovannoni G., Butzkueven H., Dhib-Jalbut S., Hobart J., Kobelt G., Pepper G., Sormani M.P., Thalheim C., Traboulsee A., Vollmer T. (2016). Brain Health: Time Matters in Multiple Sclerosis. Mult. Scler. Relat. Disord..

[3] Chalah M.A., Riachi N., Ahdab R., Créange A., Lefaucheur J.P., Ayache S.S. (2015). Fatigue in Multiple Sclerosis: Neural Correlates and the Role of Non-Invasive Brain Stimulation. Front. Cell. Neurosci..

[4] Coca-Tapia M., Cuesta-Gómez A., Molina-Rueda F., Carratalá-Tejada M. (2021). Gait Pattern in People with Multiple Sclerosis: A Systematic Review. Diagnostics.

[5] Molina-Rueda F., Fernández-Vázquez D., Navarro-López V., Miangolarra-Page J.C., Carratalá-Tejada M. (2022). The Timing of Kinematic and Kinetic Parameters during Gait Cycle as a Marker of Early Gait Deterioration in Multiple Sclerosis Subjects with Mild Disability. J. Clin. Med..

[6] Benedetti M.G., Piperno R., Simoncini L., Bonato P., Tonini A., Giannini S. (1999). Gait abnormalities in minimally impaired multiple sclerosis patients. Mult. Scler. J..

[7] Pau M., Coghe G., Corona F., Marrosu M.G., Cocco E. (2015). Effect of spasticity on kinematics of gait and muscular activation in people with Multiple Sclerosis. J. Neurol. Sci..

[8] Severini G., Manca M., Ferraresi G., Caniatti L.M., Cosma M., Baldasso F., Straudi S., Morelli M., Basaglia N. (2017). Evaluation of Clinical Gait Analysis parameters in patients affected by Multiple Sclerosis: Analysis of kinematics. Clin. Biomech..

[9] Huisinga J.M., Schmid K.K., Filipi M.L., Stergiou N. (2013). Gait mechanics are different between healthy controls and patients with multiple sclerosis. J. Appl. Biomech..

[10] Sosnoff J.J., Sandroff B., Motl R.W. (2012). Quantifying gait abnormalities in persons with multiple sclerosis with minimal disability. Gait Posture.

[11] Kempen J.C., Doorenbosch C.A., Knol D.L., de Groot V., Beckerman H. (2016). Newly Identified Gait Patterns in Patients With Multiple Sclerosis May Be Related to Push-off Quality. Phys. Ther..

[12] Lencioni T., Jonsdottir J., Cattaneo D., Crippa A., Gervasoni E., Rovaris M., Bizzi E., Ferrarin M. (2016). Are Modular Activations Altered in Lower Limb Muscles of Persons with Multiple Sclerosis during Walking? Evidence from Muscle Synergies and Biomechanical Analysis. Front. Hum. Neurosci..

[13] Smith A.M. (1981). The coactivation of antagonist muscles. Can. J. Physiol. Pharmacol..

[14] Hortobágyi T., Devita P. (2006). Mechanisms responsible for the age-associated increase in coactivation of antagonist muscles. Exerc. Sport Sci. Rev..

[15] Lang K.C., Hackney M.E., Ting L.H., McKay J.L. (2019). Antagonist muscle activity during reactive balance responses is elevated in Parkinson’s disease and in balance impairment. PLoS ONE.

[16] Keloth S.M., Arjunan S.P., Raghav S., Kumar D.K. (2021). Muscle activation strategies of people with early-stage Parkinson’s during walking. J. NeuroEng. Rehabil..

[17] Latash M.L. (2018). Muscle coactivation: Definitions, mechanisms, and functions. J. Neurophysiol..

[18] Busse M.E., Wiles C.M., van Deursen R.W. (2006). Co-activation: Its association with weakness and specific neurological pathology. J. Neuroeng. Rehabil..

[19] Ervilha U.F., Graven-Nielsen T., Duarte M. (2012). A simple test of muscle coactivation estimation using electromyography. Braz. J. Med. Biol. Res..

[20] Iwamoto Y., Takahashi M., Shinkoda K. (2017). Differences of muscle co-contraction of the ankle joint between young and elderly adults during dynamic postural control at different speeds. J. Physiol. Anthropol..

[21] Tramonti C., Imperatori L.S., Fanciullacci C., Lamola G., Lettieri G., Bernardi G., Cecchetti L., Ricciardi E., Chisari C. (2019). Predictive Value of Electroencephalography Connectivity Measures for Motor Training Outcome in Multiple Sclerosis: An Observational Longitudinal Study. Eur. J. Phys. Rehabil. Med..

[22] Boudarham J., Hameau S., Zory R., Hardy A., Bensmail D., Roche N. (2016). Coactivation of Lower Limb Muscles during Gait in Patients with Multiple Sclerosis. PLoS ONE.

[23] Thompson A.J., Banwell B.L., Barkhof F., Carroll W.M., Coetzee T., Cohen J.A., Fazecas F., Filippi M., Freedman M., Fujihara K. (2018). Diagnosis of multiple sclerosis: 2017 revisions of the McDonald criteria. Lancet Neurol..

[24] Kurtzke J.F. (1983). Rating neurologic impairment in multiple sclerosis: An expanded disability status scale (EDSS). Neurology.

[25] von Elm E., Altman D.G., Egger M., Pocock S.J., Gøtzsche P.C., Vandenbroucke J.P., STROBE Initiative (2008). The Strengthening the Reporting of Observational Studies in Epidemiology (STROBE) statement: Guidelines for reporting observational studies. J. Clin. Epidemiol..

[26] Hermens H.J., Freriks B., Disselhorst-Klug C., Rau G. (2000). Development of recommendations for SEMG sensors and sensor placement procedures. J. Electromyogr. Kinesiol..

[27] Gribble P.L., Mullin L.I., Cothros N., Mattar A. (2003). Role of cocontraction in arm movement accuracy. J. Neurophysiol..

[28] Perry J. (2010). Gait Analysis: Normal and Pathological Function. J. Sports Sci. Med..

[29] Numpy.org. https://numpy.org/doc/stable/reference/generated/numpy.trapz.html.

[30] Rose W. Mathematics and Signal Processing for Biomechanics Electromyogram analysis. https://www1.udel.edu/biology/rosewc/kaap686/notes/EMG%20analysis.pdf/.

[31] De Luca C.J., Gilmore L.D., Kuznetsov M., Roy S.H. (2010). Filtering the surface EMG signal: Movement artifact and baseline noise contamination. J. Biomech..

[32] Halki M., Ginn K., Naik R.G. (2012). Normalization of EMG Signals: To Normalize or Not to Normalize and What to Normalize to?. Computational Intelligence in Electromyography Analysis.

[33] Institut Hospital del Mar d’Investigacions Mèdiques. “Granmo.”. https://www.imim.es/ofertadeserveis/software-public/granmo/.

[34] Nogueira L.A.C., Dos Santos L.T., Sabino P.G., Alvarenga R.M.P., Santos Thuler L.C. (2013). Factors for lower walking speed in persons with multiple sclerosis. Mult. Scler. Int..

[35] Theunissen K., Plasqui G., Boonen A., Brauwers B., Timmermans A., Meyns P., Meijer K., Feys P. (2021). The Relationship Between Walking Speed and the Energetic Cost of Walking in Persons With Multiple Sclerosis and Healthy Controls: A Systematic Review. Neurorehabilit. Neural Repair.

[36] Higginson J.S., Zajac F.E., Neptune R.R., Kautz S.A., Delp S.L. (2006). Muscle contributions to support during gait in an individual with post-stroke hemiparesis. J. Biomech..

[37] Frigo C., Crenna P. (2009). Multichannel SEMG in clinical gait analysis: A review and state-of-the-art. Clin. Biomech..

[38] Silva A., Sousa A.S., Tavares J.M.R., Tinoco A., Santos R., Sousa F. (2012). Ankle dynamic in stroke patients: Agonist vs. antagonist muscle relations. Somatosens. Mot. Res..

[39] Janshen L., Santuz A., Arampatzis A. (2021). Muscle Synergies in Patients with Multiple Sclerosis Reveal Demand-Specific Alterations in the Modular Organization of Locomotion. Front. Hum. Neurosci..

[40] Socie M.J., Motl R.W., Pula J.H., Sandroff B.M., Sosnoff J.J. (2013). Gait variability and disability in multiple sclerosis. Gait Posture.

[41] Flegel M., Knox K., Nickel D. (2012). Step-length variability in minimally disabled women with multiple sclerosis or clinically isolated syndrome. Int. J. MS Care.

[42] Lamontagne A., Malouin F., Richards C.L. (2001). Locomotor-specific measure of spasticity of plantarflexor muscles after stroke. Arch. Phys. Med. Rehabil..

[43] Rinaldi M., Ranavolo A., Conforto S., Martino G., Draicchio F., Conte C., Varrecchia T., Bini F., Casali C., Pierelli F. (2017). Increased lower limb muscle coactivation reduces gait performance and increases metabolic cost in patients with hereditary spastic paraparesis. Clin. Biomech..

[44] Lamontagne A., Richards C.L., Malouin F. (2000). Coactivation during gait as an adaptive behavior after stroke. J. Electromyogr. Kinesiol..

[45] Sebastião E., Bollaert R.E., Hubbard E.A., Motl R.W. (2018). Gait variability and energy cost of overground walking in persons with multiple sclerosis: A cross-sectional study. Am. J. Phys. Med. Rehabil..

[46] Marvi-Esfahani M., Karimi M.T., Etemadifar M., Fatoye F. (2016). Comparison of energy consumption in different clinical forms of multiple sclerosis with normal subjects (cohort study). Mult. Scler. Relat. Disord..

[47] Buoite Stella A., Morelli M.E., Giudici F., Sartori A., Manganotti P., di Prampero P.E. (2020). Comfortable Walking Speed and Energy Cost of Locomotion in Patients with Multiple Sclerosis. Eur. J. Appl. Physiol..

[48] Cofré Lizama L.E., Strik M., Van der Walt A., Kilpatrick T.J., Kolbe S.C., Galea M.P. (2022). Gait stability reflects motor tracts damage at early stages of multiple sclerosis. Mult. Scler. J..

